# Supplementary material for: A history-dependent integrase recorder of plant gene expression with single-cell resolution
Source: Nat Commun. 2024 Oct 30;15:9362. doi: 10.1038/s41467-024-53716-1 (PMC11522408; doi:10.1038/s41467-024-53716-1)
Supplement: Supplementary file 1 — Supplementary Information [file 41467_2024_53716_MOESM1_ESM.pdf]

A history-dependent integrase recorder of plant gene expression with single cell resolution

Maranas *et al.*

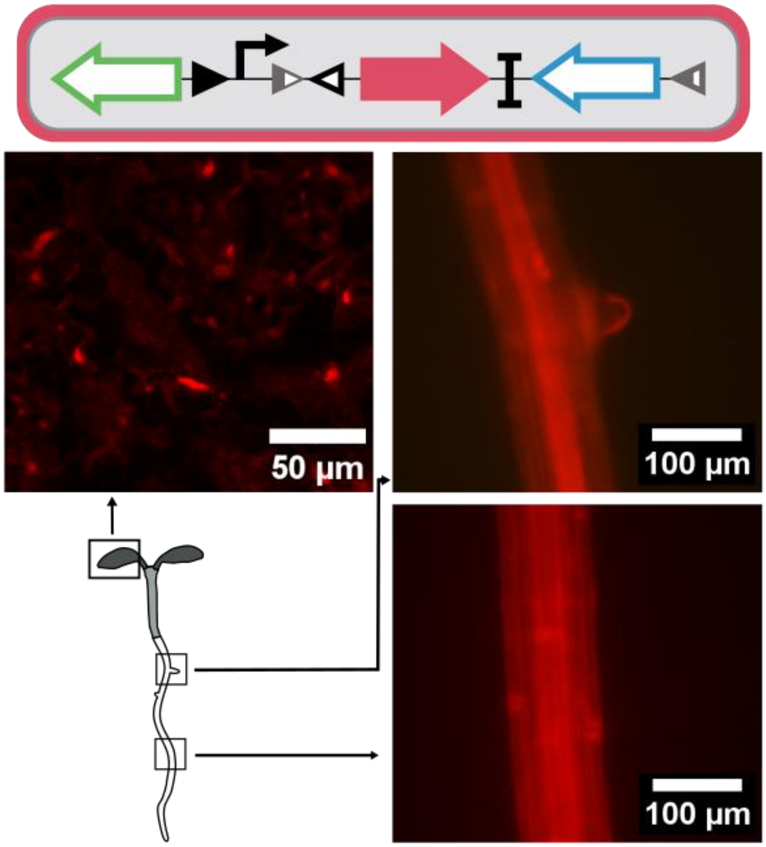

Supplementary Figure 1. Preswitched 'PhiC31 then Bxb1' integrase target circuit shows strong fluorescent expression in the leaf and root. The preswitched target was designed to test the State 1 to State 2 switch in the 'PhiC31 then Bxb1' target such that the target construct is initially in State 1, expressing RFP as per the shown target construct schematic (top). Representative leaf and root images of a T2 seedling transformed with the preswitched target (bottom).

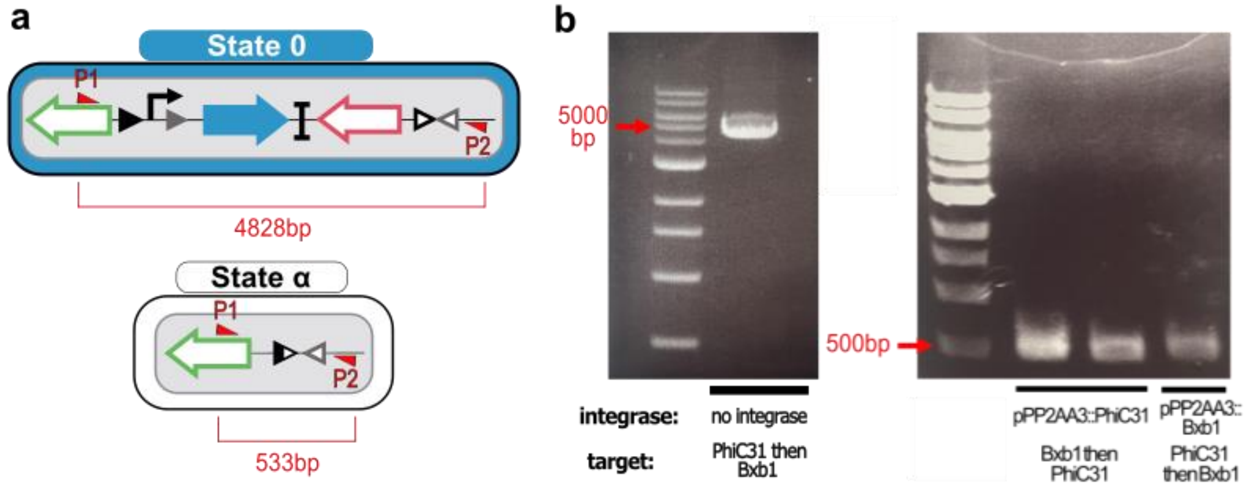

Supplementary Figure 2. State α genotyping. a Schematics of the primer binding on the full length target (shown as State 0, but will be the same size for State 1 or 2) and of the State α target. The PCR of the full length target would result in a band length of 4828 bp whereas PCR of the excised target would result in a band size of 533 bp. **b** Genotyping results. (left) Control PCR for the 'PhiC31 then Bxb1' full length target in State 0. (right) Genotyping results for pPP2AA3::PhiC31 in the 'Bxb1 then PhiC31' target (Supplementary Figure 3) (left two lanes, corresponding to two different T1 seedlings) and pPP2AA3::Bxb1 in the 'PhiC31 then Bxb1' target (right lane, one T1 seedling). Both sets of genotyping were performed twice with replicable results. Source data are provided as a Source Data file.

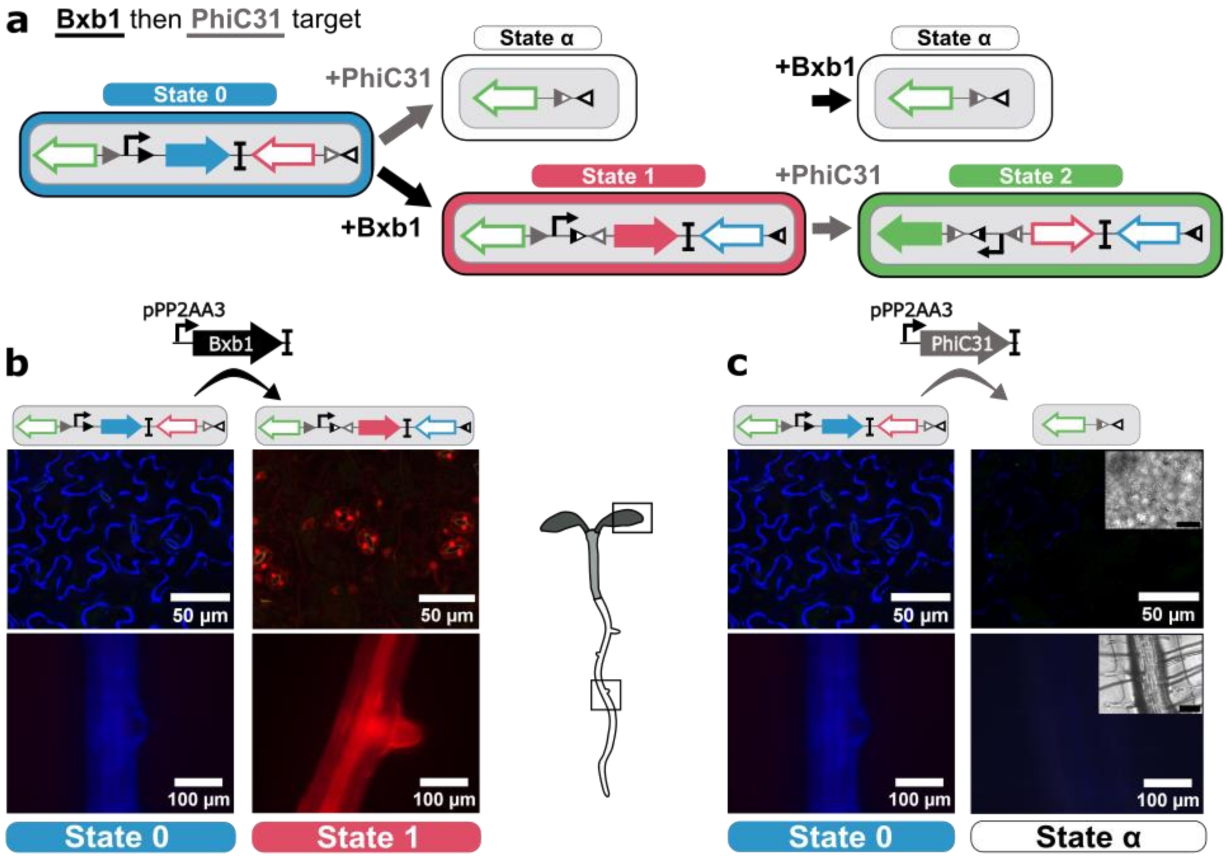

Supplementary Figure 3. The reverse order history dependent integrase circuit switches between fluorescent states. a Schematic of integrase target circuit. The design of the target is the same as in Fig. 1, but Bxb1 mediates the switch from State 0 to 1 and PhiC31 mediates the switch from State 1 to 2. Addition of the PhiC31 integrase to the State 0 target configuration results in a DNA excision and a loss of fluorescence (State α). **b** The Bxb1 integrase mediates switch from State 0 to State 1. (left) The initial target in State 0 shows strong BFP mtagBFP2 expression in the leaf and the root. (middle) Addition of constitutive Bxb1 expression to the target mediates a switch to State 1 and strong RFPmCherry fluorescence in leaf and root tissue. **c** Addition of PhiC31 prior to Bxb1 mediates an 'out of order' switch to State α and loss of fluorescence. (left) The initial target in State 0 shows strong BFPmtagBFP2 expression in the leaf and the root. (right) Addition of constitutively expressed PhiC31 to the target in State 0 results in a switch to State α and a loss in fluorescence in the leaf and root. Root and leaf tissue from 10 seedlings each from at least 2 T1 lines for each of State 0, 1, 2, and α were characterized. n=10 seedlings were screened per T1 line with at least 2 T1 lines per each of State 0, 1, and α. Black scale bars in the brightfield images correspond to 100 µm (root) and 50 µm (leaf).

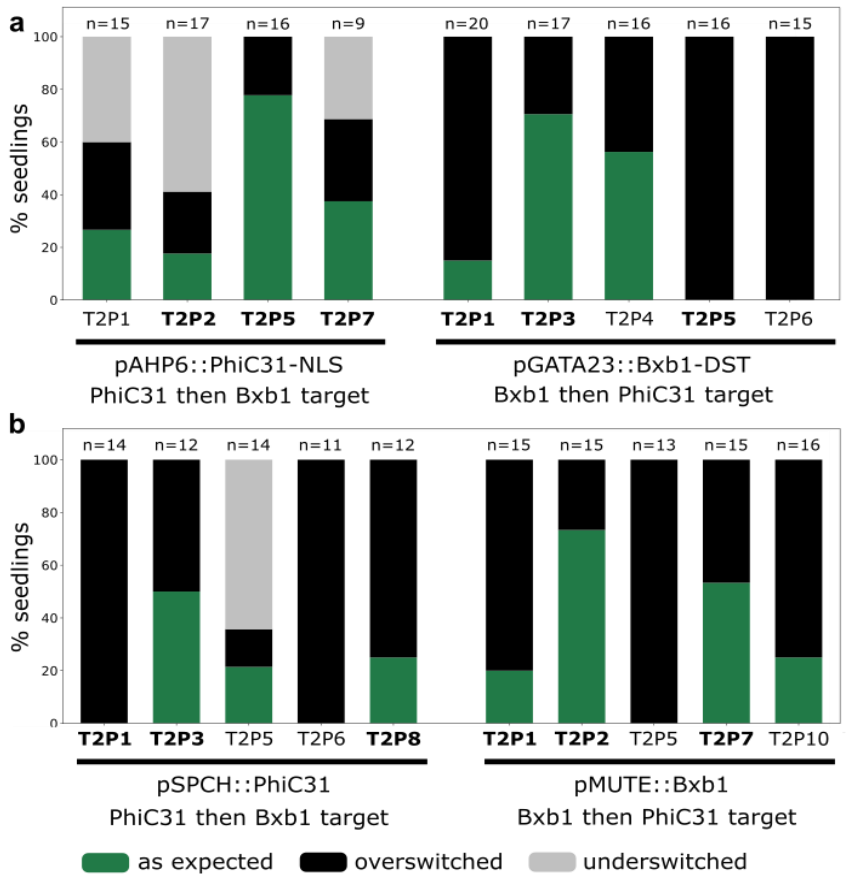

Supplementary Figure 4. Single integrase switch characterization for lateral root and stomata related genes. The bar graph corresponds to the percentage of seedling per T1 line in each category: 'as expected' (in green), overswitched (in black), and underswitched (in light grey). **a** Single integrase switch performance for lateral root genes. Seedlings were screened from T1 lines which came from T1 plants with an 'as expected' or underswitched switch pattern. The 'PhiC31 then Bxb1' target was transformed with the pAHP6::PhiC31-NLS integrase construct (left). The reverse order 'Bxb1 then PhiC31' target (Supplementary Figure 3) was transformed with the pGATA23::Bxb1-DST integrase construct (right). The number of screened seedlings for each T1 line are indicated above the bars. The bolded T1 lines correspond to the lines shown in Figures 2a and 2b. **b** Single integrase switch performance for stomata genes. Seedlings were screened from T1 lines which came from T1 plants with an 'as expected' or underswitched switch pattern. The 'PhiC31 then Bxb1' target was transformed with the pSPCH::PhiC31 integrase construct (left). The 'Bxb1 then PhiC31' target (Supplementary Figure 3) was transformed with the pGATA23::Bxb1 integrase construct (right). The number of screened seedlings for each T1 line are indicated above the bars. The bolded T1 lines correspond to the lines shown in Figures 3a and 3b. Source data are provided as a Source Data file.

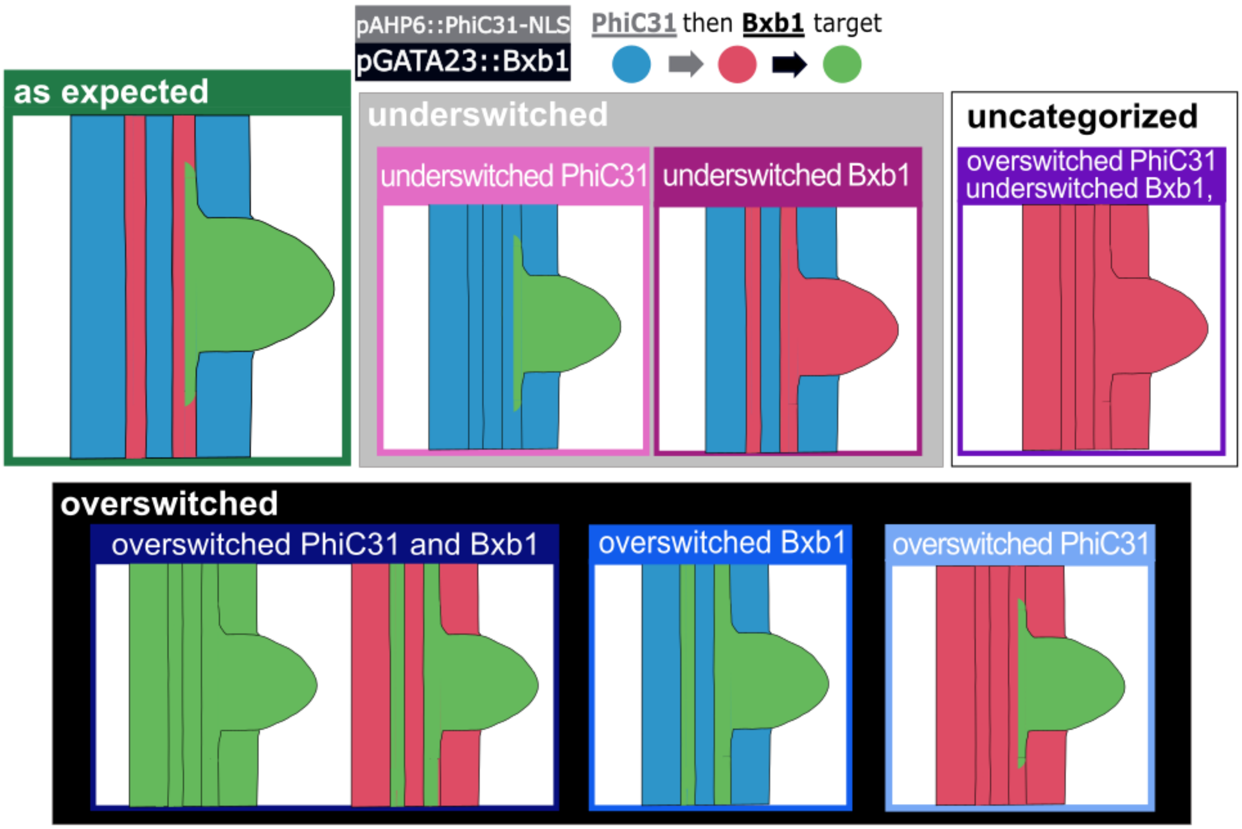

Supplementary Figure 5. Schematics for observed lateral root recorder output. The diagrams represent the lateral root recorder outputs observed for the ‘PhiC31 then Bxb1’ target transformed with the pAHP6::PhiC31-NLS-pGATA23::Bxb1 integrase construct. The box colors correspond to the key in Supplementary Figure 5 and the background boxes correspond to the categories in Figure 2.

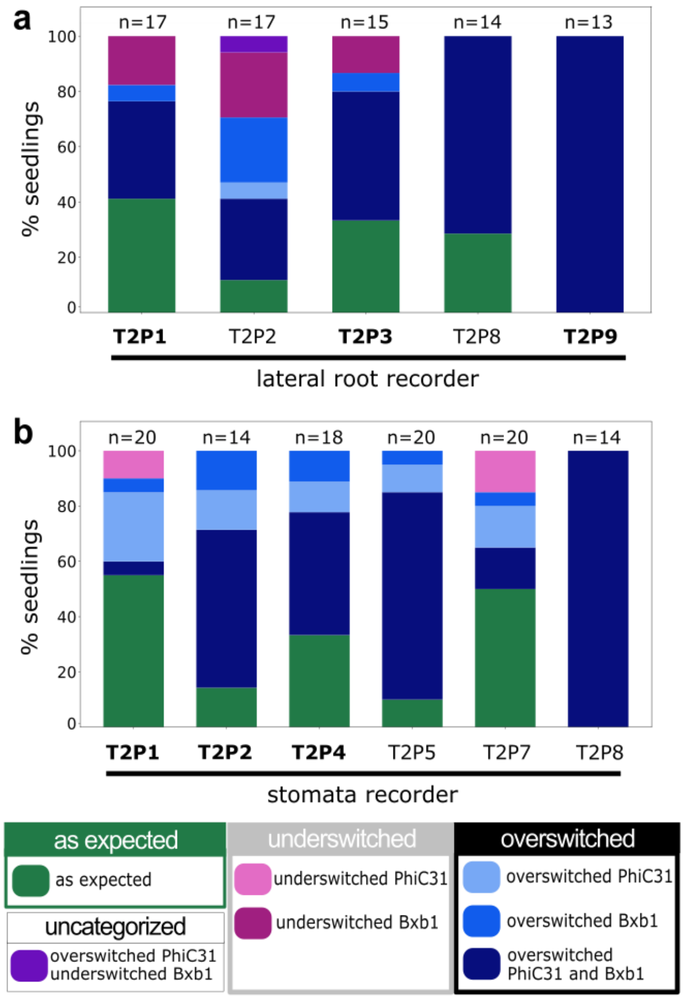

Supplementary Figure 6. Detailed characterization of recorder performance. The bar graph corresponds to the percentage of seedling per T1 line in each category. **a** Lateral root recorder performance. Seedlings were screened from 5 T2 seedlings from T1 lines which came from T1 plants with an ‘as expected’ or underswitched recorder output. The ‘PhiC31 then Bxb1’ target was transformed with the pAHP6::PhiC31-NLS-pGATA23::Bxb1 dual integrase construct. The number of seedlings screened for each T1 line is indicated above the bars. Bolded T1 lines correspond to the lines shown in Fig. 2d. The detailed switch categories correspond to the representations in Supplementary Figure 6. **b** Stomata recorder performance. Seedlings were screened from 6 T1 lines which came from T1 plants with an ‘as expected’ or underswitched recorder output. The ‘PhiC31 then Bxb1’ target was transformed with the pSPCH::PhiC31-pMUTE::Bxb1 dual integrase construct. The number of seedlings screened for each T1 line is indicated above the bars. Bolded T1 lines correspond to the lines shown in Fig 3d. The detailed switch categories correspond to the representations in Supplementary Figure 7. Source Data are provided as a Source Data file.

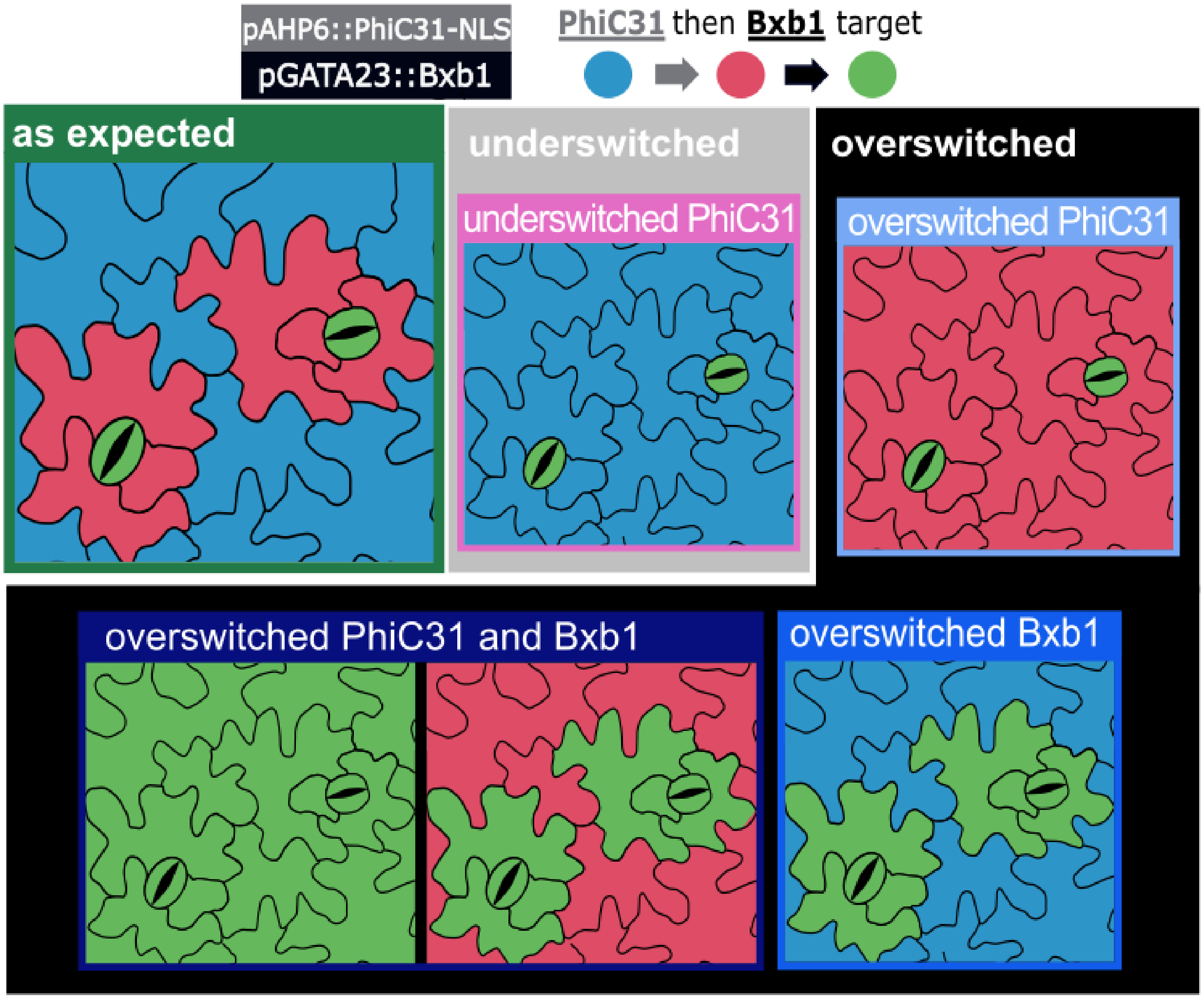

Supplementary Figure 7. Schematics for observed stomata recorder output. The diagrams represent the lateral root recorder outputs observed for the 'PhiC31 then Bxb1' target transformed with the pSPCH::PhiC31-pMUTE::Bxb1 integrase construct. The box colors correspond to the key in Supplementary Figure 5 and the background boxes correspond to the categories in Figure 3.

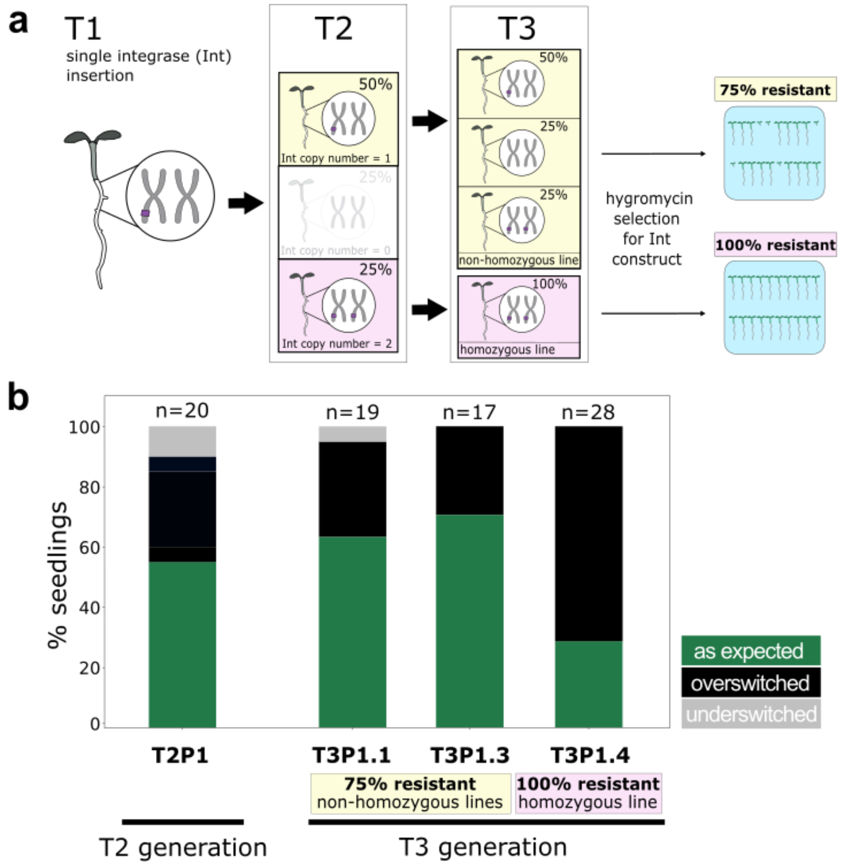

Supplementary Figure 8. Characterization of stomatal recorder in homozygous and non-homozygous T3 lines. a Schematic of integrase construct heredity from the T1 to T3 generation. The integrase construct exists as a single copy in the T1 plant (inserted via floral dip). Plants in the resulting T2 line have a mix of integrase copy numbers (one: 50%, two: 25%, zero: 25%). Plants within T3 lines descending from a homozygous parent will all be homozygous for the integrase, while plants within T3 lines descending from a heterozygous parent will show the same distribution of integrase copy number as the parental T2 line. Zygosity can be assessed in T3 via hygromycin selection wherein homozygous lines will be 100% resistant and non-homozygous lines will be 75% resistant. **b** Results of T3 characterization and hygromycin selection. Seedlings were screened from T3 lines which came from the best performing stomata recorder T2 line (T2P1, plotted for reference on the left). Only the T3 lines which showed switching were included. The number of seedlings screened for each line is indicated above the bars. Of the three T3 lines, two of them (T3P1.1 and T3P1.3) are non-homozygous (75% hygromycin resistant) and one (T3P1.4) is homozygous (100% hygromycin resistant). The 'no switch' seedlings are represented as corresponding to the seedlings which have lost the integrase construct. Source data is provided as a Source Data file.

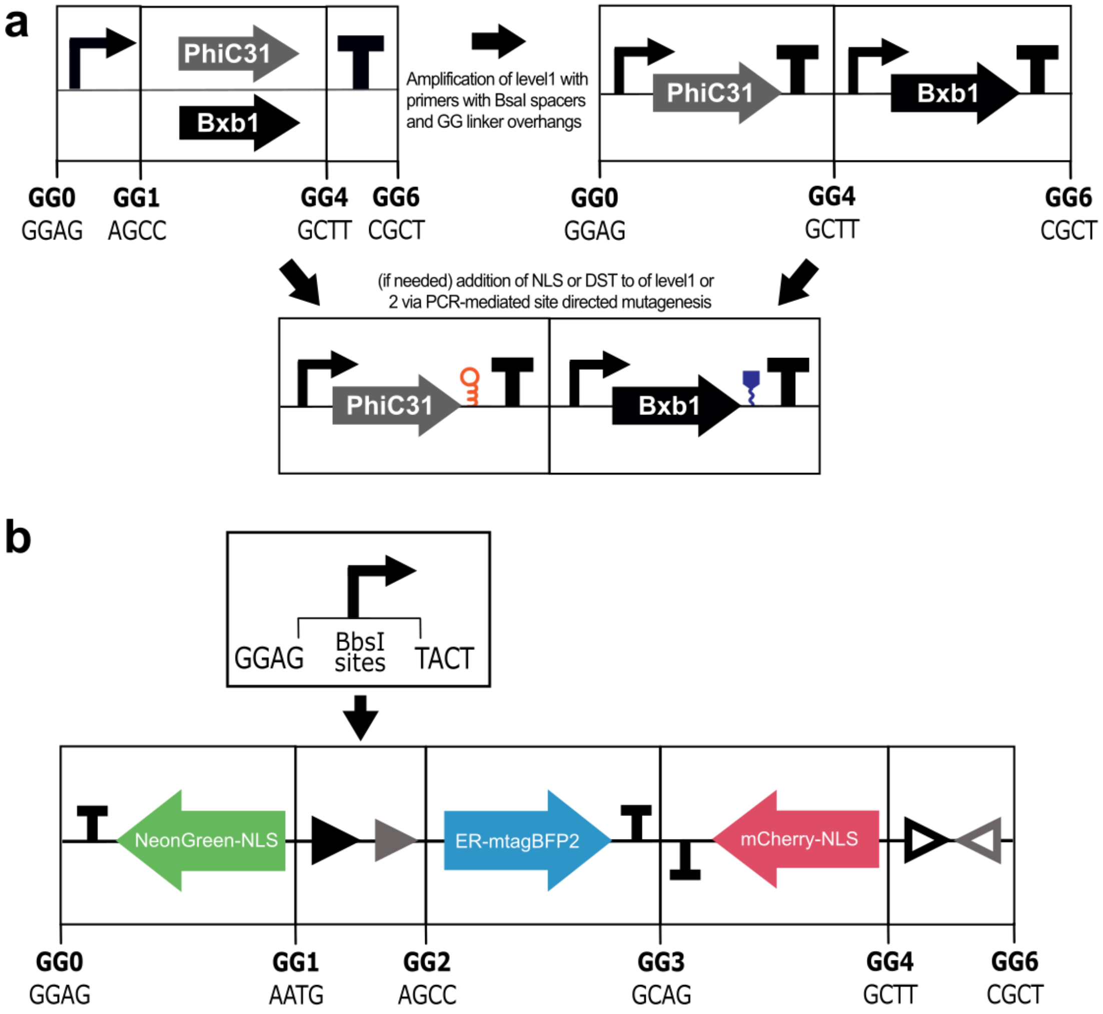

Supplementary Figure 9. Cloning strategy. a Cloning strategy for the integrase level 1 and level 2 constructs. The level 1 is built using golden gate cloning with BsaI restriction sites. The level 1 integrase constructs are then amplified using primers with overhangs including a BsaI restriction site and the appropriate GG sticky end. Then the level 2 is assembled using golden gate assembly with BsaI. If desired, tuning parts (NLS, DST) are added to the level 1 or level 2 via PCR-mediated site directed mutagenesis. **b** Cloning strategy for the target construct. The part with the integrase sites and fluorescent proteins is constructed by golden gate assembly with the BsaI enzyme. The promoter is added to the synthetic fragment via golden gate cloning using BbsI restriction sites to form the completed target.

Supplementary Table 1. Detailed overview of integrase lines and characterization categories.

	Integrase construct name	PhiC31 construct	Bxb1 construct	Target construct	As expected	Underswitched	Overswitched
pAHP6 single switch	L1P137	pAHP6::PhiC31-NLS:tUBQ1	n/a	PhiC31 then Bxb1 target (Fig. 1)	RFP in xylem and lateral roots BFP otherwise	RFP in less cells than xylem and LR	RFP in more cells than xylem and LR
pGATA23 single switch	L1B42	n/a	pGATA23::Bxb1-DST:tUBQ10	Bxb1 then PhiC31 target (Supplementary Figure 3)	RFP in LR BFP otherwise	RFP in less cells than LR	RFP in more cells than LR
Lateral root history-dependent tracker	L1PB10	pAHP6::PhiC31-NLS:tUBQ1	pGATA23::Bxb1-DST:tUBQ10	PhiC31 then Bxb1 target (Fig. 1)	GFP in LR RFP in xylem BFP otherwise	GFP in less cells than LR and/or RFP in less cells than xylem	GFP in more cells than LR and/or RFP in more cells than xylem and LR
pSPCH single switch	L1P139	pSPCH::PhiC31:tUBQ1	n/a	PhiC31 then Bxb1 target (Fig. 1)	RFP in guard cells and surrounding epidermal cells BFP otherwise	RFP in less cells than guard cells and surrounding epidermal cells	RFP in more cells than guard cells and surrounding epidermal cells
pMUTE single switch	L1B39	n/a	pMUTE::Bxb1:tUBQ10	Bxb1 then PhiC31 target (Supplementary Figure 3)	RFP in guard cells BFP otherwise	RFP in less cells than guard cells	RFP in more cells than guard cells
Stomatal history-dependent tracker	L1PB1	pSPCH::PhiC31:tUBQ1	pMUTE::Bxb1:tUBQ10	PhiC31 then Bxb1 target (Fig. 1)	GFP in guard cells RFP in surrounding epidermal cells BFP otherwise	GFP in less cells than guard cells and/or RFP in less cells than surrounding epidermal cells	GFP in more cells than guard cells and/or RFP in more cells than surrounding epidermal cells

Supplementary Table 2. Characterization of single integrase switches for lateral root genes in the T1 generation.

Tracked gene	Integrase construct	T1 seedling	Switch categorization
AHP6	pAHP6::PhiC31-NLS	T1P1	Overswitched
AHP6	pAHP6::PhiC31-NLS	T1P2	As expected
AHP6	pAHP6::PhiC31-NLS	T1P3	As expected
AHP6	pAHP6::PhiC31-NLS	T1P4	Overswitched
AHP6	pAHP6::PhiC31-NLS	T1P5	Underswitched
AHP6	pAHP6::PhiC31-NLS	T1P6	Overswitched
AHP6	pAHP6::PhiC31-NLS	T1P7	As expected
AHP6	pAHP6::PhiC31	T1P1	Underswitched
AHP6	pAHP6::PhiC31	T1P2	Underswitched
AHP6	pAHP6::PhiC31	T1P3	Underswitched
AHP6	pAHP6::PhiC31	T1P4	Underswitched
GATA23	pGATA23::Bxb1	T1P1	Overswitched
GATA23	pGATA23::Bxb1	T1P2	Overswitched
GATA23	pGATA23::Bxb1	T1P3	Overswitched
GATA23	pGATA23::Bxb1	T1P4	Overswitched
GATA23	pGATA23::Bxb1	T1P5	Overswitched
GATA23	pGATA23::Bxb1	T1P6	Overswitched
GATA23	pGATA23::Bxb1	T1P7	Overswitched
GATA23	pGATA23::Bxb1	T1P8	Overswitched
GATA23	pGATA23::Bxb1	T1P9	Overswitched
GATA23	pGATA23::Bxb1	T1P10	Overswitched
GATA23	pGATA23::Bxb1-DST	T1P1	As expected
GATA23	pGATA23::Bxb1-DST	T1P2	Overswitched
GATA23	pGATA23::Bxb1-DST	T1P3	As expected
GATA23	pGATA23::Bxb1-DST	T1P4	As expected
GATA23	pGATA23::Bxb1-DST	T1P5	As expected
GATA23	pGATA23::Bxb1-DST	T1P6	As expected
GATA23	pGATA23::Bxb1-DST	T1P7	Overswitched
GATA23	pGATA23::Bxb1-DST	T1P8	Overswitched

Supplementary Table 3. Characterization of the lateral root and stomatal recorder in the T1 generation.

Cell lineage	Tracked genes	PhiC31 construct	Bxb1 construct	T1 seedling	Switch categorization
lateral root	*AHP6, GATA23*	pAHP6::PhiC31-NLS	pGATA23::Bxb1	T1P1	Underswitched PhiC31
lateral root	*AHP6, GATA23*	pAHP6::PhiC31-NLS	pGATA23::Bxb1	T1P2	Underswitched PhiC31
lateral root	*AHP6, GATA23*	pAHP6::PhiC31-NLS	pGATA23::Bxb1	T1P3	As expected
lateral root	*AHP6, GATA23*	pAHP6::PhiC31-NLS	pGATA23::Bxb1	T1P4	Overswitched PhiC31 and Bxb1
lateral root	*AHP6, GATA23*	pAHP6::PhiC31-NLS	pGATA23::Bxb1	T1P5	Overswitched PhiC31 and Bxb1
lateral root	*AHP6, GATA23*	pAHP6::PhiC31-NLS	pGATA23::Bxb1	T1P6	Overswitched PhiC31 and Bxb1
lateral root	*AHP6, GATA23*	pAHP6::PhiC31-NLS	pGATA23::Bxb1	T1P7	Overswitched PhiC31 and Bxb1
lateral root	*AHP6, GATA23*	pAHP6::PhiC31-NLS	pGATA23::Bxb1	T1P8	Underswitched PhiC31
lateral root	*AHP6, GATA23*	pAHP6::PhiC31-NLS	pGATA23::Bxb1	T1P9	As expected
stomata	*SPCH, MUTE*	pSPCH::PhiC31	pMUTE::Bxb1	T1P1	As expected
stomata	*SPCH, MUTE*	pSPCH::PhiC31	pMUTE::Bxb1	T1P2	As expected
stomata	*SPCH, MUTE*	pSPCH::PhiC31	pMUTE::Bxb1	T1P3	Overswitched Bxb1
stomata	*SPCH, MUTE*	pSPCH::PhiC31	pMUTE::Bxb1	T1P4	As expected
stomata	*SPCH, MUTE*	pSPCH::PhiC31	pMUTE::Bxb1	T1P5	As expected
stomata	*SPCH, MUTE*	pSPCH::PhiC31	pMUTE::Bxb1	T1P6	Overswitched Bxb1
stomata	*SPCH, MUTE*	pSPCH::PhiC31	pMUTE::Bxb1	T1P7	As expected
stomata	*SPCH, MUTE*	pSPCH::PhiC31	pMUTE::Bxb1	T1P8	As expected
stomata	*SPCH, MUTE*	pSPCH::PhiC31	pMUTE::Bxb1	T1P9	Overswitched Bxb1

Supplementary Table 4. Characterization of single integrase switches for stomatal genes in the T1 generation.

**Tracked gene**	**Integrase construct**	**T1 seedling**	**Switch categorization**
SPCH	pSPCH::PhiC31	T1P1	As expected
SPCH	pSPCH::PhiC31	T1P2	Overswitched
SPCH	pSPCH::PhiC31	T1P3	As expected
SPCH	pSPCH::PhiC31	T1P4	Overswitched
SPCH	pSPCH::PhiC31	T1P5	Underswitched
SPCH	pSPCH::PhiC31	T1P6	As expected
SPCH	pSPCH::PhiC31	T1P7	Overswitched
SPCH	pSPCH::PhiC31	T1P8	As expected
SPCH	pSPCH::PhiC31	T1P9	Overswitched
SPCH	pSPCH::PhiC31	T1P10	Overswitched
MUTE	pMUTE::Bxb1	T1P1	As expected
MUTE	pMUTE::Bxb1	T1P2	As expected
MUTE	pMUTE::Bxb1	T1P3	Overswitched
MUTE	pMUTE::Bxb1	T1P4	Overswitched
MUTE	pMUTE::Bxb1	T1P5	As expected
MUTE	pMUTE::Bxb1	T1P6	Overswitched
MUTE	pMUTE::Bxb1	T1P7	As expected
MUTE	pMUTE::Bxb1	T1P8	Overswitched
MUTE	pMUTE::Bxb1	T1P9	Overswitched
MUTE	pMUTE::Bxb1	T1P10	As expected
