# Supplementary material for: A history-dependent integrase recorder of plant gene expression with single-cell resolution
Source: Nat Commun. 2024 Oct 30;15:9362. doi: 10.1038/s41467-024-53716-1 (PMC11522408; doi:10.1038/s41467-024-53716-1)
Supplement: Supplementary file 3 — Description of Additional Supplementary Files [file 41467_2024_53716_MOESM3_ESM.pdf]

**Description of Additional Supplementary Files**

File Name: Supplementary  Data 1
Description: List of oligos used in this study. This file includes id number, name, sequence, and purpose for each oligo used in this study.

File Name: Supplementary Data 2
Description: List of constructs used in this study. This file lists for each level 0 construct in this study: the construct short name, detailed name, golden gate linkers, primers used to generate the level 0 (if applicable), method of construction, addgene deposit number, bacterial antibiotic resistance, and full sequence. For level 1 constructs used in this study the file lists the short name, detailed name, golden gate assembly scheme, addgene deposit number, bacterial antibiotic resistance, plant antibiotic resistance, and full sequence. For each available seed line, the ABRC stock number is also provided in this file.
